# Exploring the Mechanisms Underlying Drug-Induced Fractures Using the Japanese Adverse Drug Event Reporting Database

**DOI:** 10.3390/ph14121299

**Published:** 2021-12-13

**Authors:** Shinya Toriumi, Akinobu Kobayashi, Hitoshi Sueki, Munehiro Yamamoto, Yoshihiro Uesawa

**Affiliations:** 1 Department of Medical Molecular Informatics, Meiji Pharmaceutical University, Kiyose 204-8588, Japan; 2 Department of Pharmacy, National Hospital Organization Kanagawa Hospital, Hadano 257-8585, Japan; kobayashi.akinobu.tk@mail.hosp.go.jp; 3 Department of Orthopedic Surgery, National Hospital Organization Kanagawa Hospital, Hadano 257-8585, Japan; hitoshi.sueki@gmail.com (H.S.); mohey-mohey@kanagawa-hosp.org (M.Y.)

**Keywords:** drug-induced fracture, atypical femoral fracture, bisphosphonates, denosumab, spontaneous reporting system, Japanese Adverse Drug Event Report (JADER), pharmacovigilance, volcano plot, multiple logistic regression analysis, principal component analysis

## Abstract

Fractures occur when bones become fragile and are subjected to external forces as occurring during falls. The use of drugs that increase bone fragility or fall risk increases the risk of fracture. This study investigates drug-induced fractures reported in the Japanese Adverse Drug Event Report (JADER) database in patients using 4892 drugs. Atypical femur fracture was the most frequently reported fracture, and 58 other fractures were also reported. Using Volcano plots and multiple logistic regression analysis, we identified the risk factors for drug-induced fractures as being female, of older age, higher body mass index, and using one of 90 drugs. The drug groups significantly associated with drug-induced fractures included bone resorption inhibitors, antiviral drugs, dopaminergic drugs, corticosteroids, and sleep sedatives. Principal component analysis was used to examine the relationship between the use of specific drugs and the site of drug-induced fracture. Bone resorption inhibitors and corticosteroids were associated with atypical femur fractures, jaw fractures, and ulna fractures through an osteoclast-mediated process. Other drugs were found to increase fracture risk via non-osteoclast-mediated mechanisms. These findings suggest that many drugs can result in drug-induced fractures through a variety of mechanisms.

## 1. Introduction

Patient hospitalization for fractures is associated with the use of a variety of medications. Whenever possible, health care providers need to consider the risk of fracture when prescribing such medications. The risk of fractures is related to bone density, bone quality, and the application of external forces as occurs during falls. Stone et al. reported that fractures at of the proximal femur, wrist, and spinal vertebrae correlate significantly with decreased bone density; however, the contribution of bone density to these fractures was only 10–44% [1]. In contrast, falls were responsible for 98% of proximal femur fractures [2], 97% of humerus fractures, and 100% of forearm fractures [3]. Thus, fracture risk is influenced by multiple factors, and drugs that act on these factors can potentially increase this risk.

Drugs can increase fracture risk through a variety of mechanisms. Adrenal corticosteroids increase the risk of fracture by causing steroidal osteoporosis [4]. Benzodiazepines and other sleep sedatives and drugs for Parkinson’s disease increase the risk of falls, thereby increasing fracture risk [5]. Drug effects on osteoclasts are involved in medication-related osteonecrosis of the jaw (MRONJ) and atypical femur fracture (AFF), which are rare side effects of bisphosphonates (BPs) and anti-receptor activator of nuclear factor-κ B ligand (RANKL) antibody [6]. Exhaustive studies examining the effects of a wide range of drugs on fracture risk and the underlying mechanisms have not been conducted, leaving the following questions unanswered: What drugs contribute to drug-related fractures? In which bones do drug-related fractures occur? What are the mechanisms underlying drug-related fractures? This study uses data in the Japanese Adverse Drug Event Report (JADER) database, a collection of spontaneously reported adverse drug reactions, to answer these clinical questions.

## 2. Results

### 2.1. Construction of Data Analysis Tables

**Figure 1:**
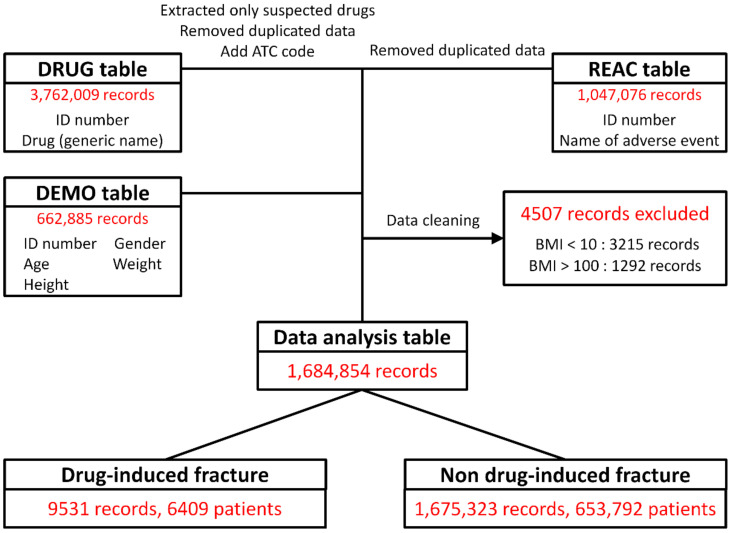
Flowchart for construction of the study cohort for data analysis. Causes of drug-related fracture for each drug in the DRUG table (drug name, causality) were classified into three categories: “suspected drug,” “concomitant drug,” and “interaction drug.” Only data in the “suspected drug” category were extracted. Data duplicated in the DRUG and REAC tables were removed [7]. Data in the DEMO table (patient characteristics such as sex, age, and weight) were combined with the DRUG and REAC tables using patient identification numbers. Cases involving patients with a body mass index (BMI) of <10 or >100 were removed.

A flowchart of the extraction of data for analysis in this study is shown in Figure 1. Data were extracted from the JADER DRUG table (3,762,009 records), REAC table (1,047,076 records), and DEMO table (662,885 records). The data in the three tables were combined, and 4507 ineligible records were deleted. The final cohort for data analysis included 1,684,854 records, of which 9531 (0.6%) were reports of drug-induced fractures.

### 2.2. Sites of Drug-Induced Fractures

Table 1 shows the 58 types of fractures reported as drug-induced fractures. The most frequently reported drug-induced fractures were AFF (1653; 17.3%), fracture (1550; 16.3%), femur fracture (1366; 14.3%), spinal compression fracture (1059; 11.3%), femoral neck fracture (619; 6.5%), compression fracture (355; 3.7%), and rib fracture (261; 2.7%).

### 2.3. Association between Patient Characteristics and Drug-Induced Fractures

The patient characteristics found to be associated with drug-induced fractures are shown in Table 2. The majority of patients in the drug-induced fracture group were female (6533; 72.1%). The mean ± standard deviation of age, height, weight, and body mass index (BMI) of the drug-induced fracture group were 69.1 ± 17.4 years, 154.0 ± 12.6 cm, 52.1 ± 13.7 kg, and 22.2 ± 4.5, respectively. The non-drug-induced fracture groups were 59.5 ± 21.5 years, 157.2 ± 18.3 cm, 54.5 ± 16.3 kg, and 21.9 ± 4.5, respectively. Univariate regression analysis showed significant differences in sex, age, height, weight, and BMI between these groups.

### 2.4. Association between Drug Use and Drug-Induced Fractures

**Figure 2:**
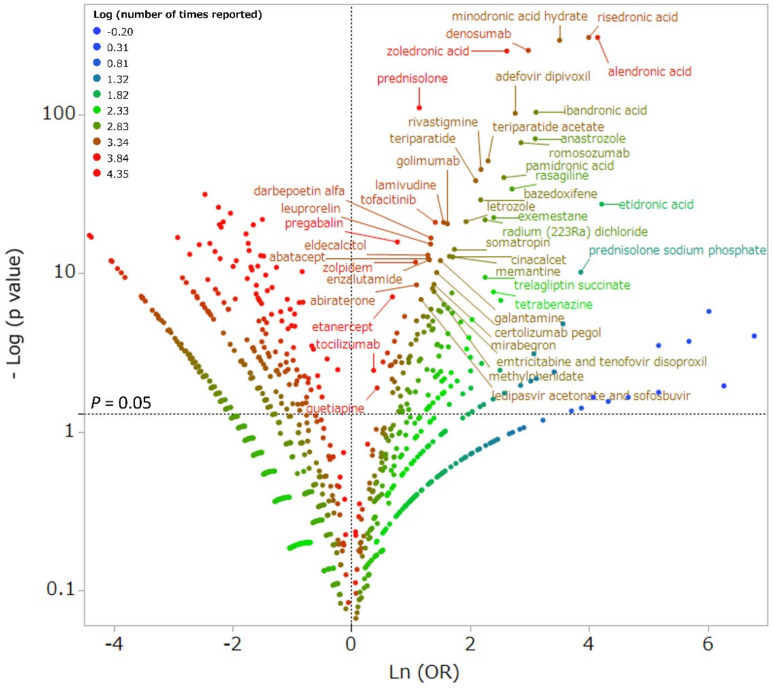
Drugs associated with drug-induced fracture. The X-axis shows the natural logarithm of the odds ratios (ln ([OR]), and the Y-axis shows the common logarithm of the inverse *p*-value (−log10 [p]) from Fisher’s exact test. The ORs were calculated using cross-tabulation. The dotted line on the Y-axis represents *p* = 0.05. Plot colors represent the number of reports of adverse events. The red-green-blue points are common logarithms of the total reported numbers (range, −0.20 to 4.35). As the ORs become more positive, the tendency toward adverse events increases; decreasing *p*-values indicates greater statistical significance. The upper-right portion of the scatter plot identifies drugs that more highly associated with drug-induced fracture.

The scatter plot in Figure 2 depicts the association between specific drugs and drug-induced fractures. Red dots indicate the drugs with the most reported drug-induced fractures. Therefore, drugs that are plotted in the upper-right corner and have a red dot have the greatest potential to cause drug-induced fractures. The most frequently reported drugs with a high potential to cause drug-induced fractures were alendronic acid (1046 reports; 11.0%), prednisolone (569 reports; 6.0%), risedronic acid (517 reports; 5.4%), zoledronic acid (353 reports; 3.7%), and denosumab (297 reports; 3.1%). The 138 drugs found to be associated with drug-induced fractures are listed in Supplementary Table S1.

### 2.5. Independent Risk Factors for Drug-Induced Fracture by Multiple Logistic Regression Analysis

Multiple logistic regression analysis showed that the independent risk factors for drug-induced fracture were being female, of older age, having a high BMI, and the administration of 90 drugs (Table 3). Among these drugs, there were 10 drugs that affect bone structure and mineralization (Anatomical Therapeutic Chemical (ATC) code: M05B). Of these, eight were bisphosphonates (etidronic acid, alendronic acid, minodronic acid, risedronic acid, ibandronic acid, incadronic acid, zoledronic acid, and pamidronic acid) and one was denosumab, an anti-RANKL antibody. There were 11 antiviral agents (ATC code: J05A), including the nucleoside nucleic acid reverse transcriptase inhibitors (NRTIs) abacavir, lamivudine, lamivudine abacavir, tenfovir disoproxil fumarate, emtricitabine tenfovir disoproxil fumarate, and ritonavir, a protease inhibitor (PI), and dolutegravir, an integrase chain transfer inhibitor, were the seven anti-human immunodeficiency virus (HIV) drugs. Seven dopaminergic drugs (ATC code: N04B) were Parkinsonian drugs: levodopa benserazide hydrochloride, levodopa carbidopa hydrate, the dopamine agonists pramipexole, ropinirole, and rotigotine, the catechol-O-methyltransferase inhibitor entacapone, and the monoamine oxidase B inhibitor rasagiline. The seven dopaminergic drugs were the catechol O-methyltransferase inhibitor entacapone and the monoamine oxidase B inhibitor rasagiline. There were five adrenocorticosteroids (ATC code: D07A): prednisolone, methylprednisolone, hydrocortisone, betamethasone, and sodium prednisolone phosphate; five hormone antagonists and related drugs (ATC code: L02B): anastrozole, exemestane, and letrozole (aromatase inhibitors), and abiraterone acetate and enzalutamide (antiandrogens); five sleeping pills and analgesics (ATC code: N05C): brotizolam, a short-acting benzodiazepine; zolpidem tartrate, zopiclone, and eszopiclone, ultra-short-acting nonbenzodiazepines; and suvorexant, an orexin receptor antagonist.

### 2.6. Association between Drug Use and the Drug-Induced Fracture Site by Principal Component Analysis

Principal component analysis showed that the first principal component contributed 48.3% and the second principal component contributed 13.0%. A scatter plot was created using the first and second principal components.

**Figure 3:**
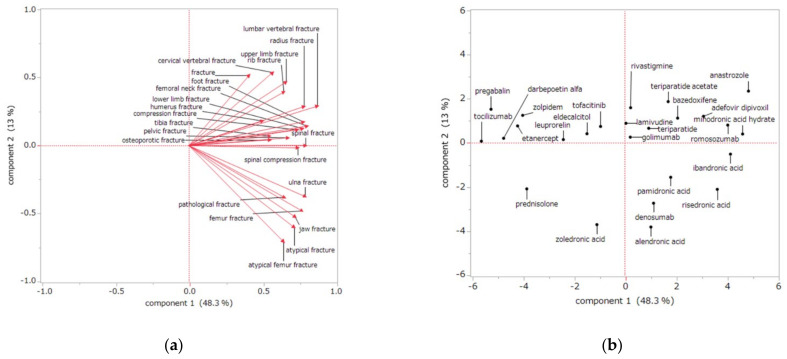
Association between the drug-induced fracture site and specific drug use according to principal component analysis. Loading vectors represent the relationship between the adverse events and principal components (**a**). Each loading vector indicates an adverse event. The score plot shows the relationships between the drugs and principal components (**b**). Each dot indicates a drug.

The relationship between drug-induced fractures and the principal components is shown in Figure 3a. Each adverse event is represented as a loading vector, with the X-axis representing the first principal component and the Y-axis representing the second principal component. All positively correlated side effect vectors are plotted on the X-axis. On the Y-axis, the adverse event vectors are plotted separately for the top and bottom. For the second principal component, each adverse event lower vector showed a large negative correlation with AFF (−0.71), atypical fracture (−0.60), jaw fracture (−0.52), femur fracture (−0.48), pathological fracture (−0.38), ulna fracture (−0.37). A large positive correlation was observed for cervical vertebral fracture (0.54), fracture (0.52), and upper limb fractures (0.47).

Figure 3b shows the relationship between drugs and their main components. BPs and denosumab, mainly used to treat osteoporosis, showed positive values in the first principal component and negative values in the second principal component. The first and second principal components of BP and prednisolone, which are mainly used to treat malignant tumors, both showed negative values. Other drugs showed positive values in the second principal component. For example, anastrozole showed positive values in the first and second principal components, and zolpidem showed a negative value for the first principal component and a positive value for the second principal component.

## 3. Discussion

### 3.1. Drug-Induced Fracture Sites

A total of 9531 fractures of 58 different types were reported as drug-induced fractures. The most commonly reported were AFFs, fractures, femur fractures, vertebral compression fractures, and femoral neck fractures (Table 1). Thus, drug-induced fractures occurred at many sites, mainly in the femur and spine.

The first report of an AFF was described by Odvina et al. in 2005 in a patient with severely suppressed bone turnover caused by the use of BPs [8]. AFFs occur in the subtrochanteric and diaphyseal regions, and the characteristic imaging findings and clinical manifestations are fatigue fractures or fragility fractures, which are clearly distinguished from typical femoral fractures [9]. Although the cause of AFFs remains unclear, Shana et al. have stated that bone resorption inhibitors such as BPs and denosumab are risk factors [9]. In addition, Ruggiero et al. reported MRONJ as a rare adverse event of BPs and denosumab [10], both of which are reported to affect osteoclasts [6]. The present study cohort included 92 reports of jaw fractures, which may include MRONJ (Table 1), and also included many reports of fractures particular to bone resorption inhibitors.

In addition to AFFs, fractures of the femur and spine were frequently reported in our cohort. These fractures are thought to be related to osteoporosis and falls. The sites of fractures frequently caused by osteoporosis include the femoral head, spine, radius, and humerus [11]. Our findings are consistent in this report, suggesting that drugs may increase fracture risk by being associated with osteoporosis and falls; femoral neck fracture is the most severe of the fractures associated with osteoporosis and can impair walking and cause patients to become bedridden, significantly reducing the quality of life [12,13]. Spinal compression fractures are the most frequent fractures associated with osteoporosis [14]. Wrist and humerus fractures can occur during falls, particularly if bone strength is reduced due to osteoporosis; for example, osteoporosis due to corticosteroids is a risk factor for fracture [15,16], and benzodiazepines increase the risk of falling and its associated fractures [17].

### 3.2. Risk Factors for Drug-Induced Fractures

Our comprehensive analysis of more than 4000 drugs suggests that drug-induced fractures are more common in those who are female, of older age, with higher BMI, and are taking one of 90 drugs. The 90 drugs associated with drug-induced fractures include many drugs from the following groups: drugs that affect bone structure and mineralization, direct acting antivirals, dopaminergic drugs, sleeping pills and sedatives, and corticosteroids (Table 3).

As people age, bone resorption exceeds bone formation in both men and women, resulting in a decrease in bone density, deterioration of bone microstructure, and a decrease in the degree of calcification that decrease bone strength [18,19]. In a Japanese survey of AFFs, the most commonly reported type of drug-induced fracture in this study, 23 of 24 AFFs occurred in women, and the average age was 73.0 years [20]. Similarly, we observed that 72.1% of the drug-induced fractures were in women, and the mean age was 69.1 years. The risk of AFF is reported to increase with high BMI [21,22]. In patients with osteoporosis, low BMI increases the risk of hip fracture, while high BMI increases the risk of upper limb fracture [23]. In our cohort of drug-induced fractures, AFFs (17.3%) and humerus fractures (2.1%) associated with high BMI were more frequent than femoral neck fractures (6.5%) associated with low BMI. Therefore, our findings suggest the possibility of screening for drug-induced fractures using patient characteristics.

In this study, 10 drugs that affect bone structure and mineralization were found to be associated with drug-induced fractures. These included eight BPs and denosumab, an anti-RANKL antibody. Used to treat osteoporosis and malignancy, BPs inhibit osteoclast activity and decrease bone resorption through their strong affinity for bone hydroxyapatite [24]. However, BPs are associated with AFF [9] and MRONJ [10], and Shana et al. showed that AFF is a risk of long-term BP administration [9]. The results of a meta-analysis showed a 28-fold increase in the relative risk of AFF with BP use [25]. In this study, our drug-related fracture group reported the use of alendronic acid (11.0%), risedronic acid (5.4%), and minodronic acid (2.9%), drugs mainly used in osteoporosis patients. Zoledronic acid, mainly used to treat for malignant tumors, was reported by 3.7%. In addition, all BPs were extracted as independent risk factors. These findings suggest that any of the BPs may put patients at risk of drug-induced fractures; we also observed that the use of denosumab, another bone resorption inhibitor [26,27], was an independent risk factor for AFF. Similar to BPs, denosumab has been reported to cause AFFs [28,29,30] and MRONJ [31]. Together with our observations, these findings suggest that denosumab should be prescribed with the same level of caution as BPs.

We found that 11 direct acting antivirals were associated with drug-induced fractures; of these, 7 were anti-HIV drugs. Although advances in anti-HIV therapy have markedly improved patient prognosis, osteoporosis and fractures are challenges associated with long-term treatment and an aging patient population. The incidence of osteoporosis is reported to be three times higher in patients with HIV than in those without [32]. Although the underlying mechanism is still unclear, HIV infection is associated with increased differentiation into osteoclasts, accelerated bone resorption due to osteoclast activation, decreased differentiation into osteoblasts, increased osteoblast apoptosis, and suppressed bone formation due to decreased osteoblast activity [33]. The risk of bone loss among HIV patients is reported to be 2.5-fold greater in those undergoing treatment than in those who are untreated [32]. Drugs implicated in bone loss among treated HIV patients include PI, NRTIs and non-NRTIs [34], as observed in the present study.

Our findings suggest that corticosteroids and hormone antagonists and related drugs are associated with drug-induced fractures. Corticosteroids are commonly used to treat inflammatory and autoimmune diseases. Long-term administration of corticosteroids is known to cause pathological bone fractures. Oral steroid use of more than 5 mg prednisolone equivalents per day decreases bone density and increases the risk of fracture [35]. After 3 to 6 months of use, the risk of fracture with corticosteroid use increases to 2–4 times that of non-users [36]. In the U.S., 20% of the 20 million osteoporosis patients are taking steroids, and fractures are estimated to occur in about half of the patients with long-term steroid use [37]. In the UK, 0.5% of the total population is treated with oral steroids, but only 14% of these patients are receiving prevention or treatment for osteoporosis [38]. The pathogenesis of steroid-induced osteoporosis includes direct effects on bone metabolism, mainly through inhibition of osteoblasts and other osteogenic cells and by indirect effects on the endocrine system. The pathophysiology of steroid-induced osteoporosis includes the promotion of osteoblast and osteocyte apoptosis [39,40] and calcium excretion in urine and the suppression of osteoclast apoptosis [41], sex hormone secretion (e.g., estrogen, testosterone) [42], and calcium absorption from the intestinal tract. In this study, five hormone antagonists and related drugs were found to be associated with drug-induced fracture. Aromatase inhibitors, which decrease blood estrogen levels, are the first choice in postoperative hormone therapy for postmenopausal breast cancer. Blood estradiol concentration is closely related to bone metabolism in postmenopausal women. Estradiol decrease correlates with bone mineral density loss [43], increasing the risk of fracture [44]. Used to treat prostate cancer, antiandrogens suppress androgen production, resulting in decreased bone density through decreased blood estrogen levels [45]. In the present study, many drugs associated with the risk of osteoporosis were found to be associated with drug-induced fractures.

Our results show that the use of sleeping pills, sedatives, and dopaminergic drugs, which can cause falls, is associated drug-induced fractures. Five types of sleeping pills and sedatives were associated with drug-induced fractures. Brotizolam and zolpidem are both benzodiazepine receptor agonists, although they differ in their selectivity for receptor subtypes. Benzodiazepine receptors in the brain are involved in hypnotic sedation, anxiolysis, and muscle relaxation, causing drowsiness, dizziness, and imbalance [46]. Previous studies, including meta-analyses, clearly show an increased risk of falls associated with the use of benzodiazepine receptor agonists [47,48]. In addition, these drugs have been shown to increase the risk of adverse falls, which correlate significantly with age and are fatal in more than 9%, especially in those over 80 years of age [49]. The short-acting benzodiazepine receptor agonists are more likely to cause mid-wake; in some cases, muscle relaxation may continue during mid-wake, presenting a risk of falls and fractures.

We also found that drug-induced fractures were associated with the use of seven dopaminergic drugs. Previous reports have been reported with levodopa and dopamine agonists [50,51,52]; levodopa provides the most improvement in Parkinsonian symptoms, but motor complications, such as the wearing-off phenomenon, may occur. Dopamine agonists are less effective at improving symptoms than levodopa and therefore have a lower risk of motor complications, but have a higher incidence of daytime hypersomnia and idiopathic sleep [53]. Therefore, sudden sleep may occur while the patient is walking, causing a fall. Although the occurrence of sudden sleep can be decreased by reducing or changing the dose of dopamine agonists [54], caution should be exercised regarding the effects on motor symptoms. Although there are few studies report an association between monoamine oxidase or catechol-O-methyltransferase inhibitors and falls or fractures, our results suggest that there may be an association.

### 3.3. Association between Drug Use and the Drug-Induced Fracture Site

Our principal component analysis findings show that the use of BPs and denosumab increase the risk of AFFs, jaw fractures, and ulna fractures. Osteoclast suppression by the use of BPs is associated with AFFs and other atypical fractures [9] and with MRONJ [10]. In the present study, a severe case of MRONJ may have been reported as a fracture of the jaw.

Of the drug-induced fractures, AFF, atypical fracture, jaw fracture, pathological fracture, and ulna fracture had similar characteristics in the loading vector (Figure 3a). These sites of drug-induced fractures correlated positively with the first principal component and negatively with the second principal component in the analysis, indicating that these fractures may be strongly influenced by osteoclasts. MRONJ and AFFs are reported to have similar characteristics [6,55]. Several studies have reported that osteoclast suppression by BPs is involved in ulnar fractures [56,57]. In our study, ulnar fractures showed similar characteristics to atypical femoral and jaw fractures, suggesting an association with osteoclasts. Therefore, patients at high risk for AFF and MRONJ should have their ulnae monitored.

Score plots (Figure 3b) of our data show that most of the BPs correlated negatively with the second principal component, suggesting that fractures caused by BP use may involve osteoclasts. Furthermore, first principal component analysis showed that the BPs alendronic acid and risedronic acid, prescribed for osteoporosis, plotted to the right of zoledronic acid, a BP used to treat malignancies. This finding suggests that BPs used for osteoporosis may contribute more to AFFs than do BPs used for malignancy. Previous reports have suggested that prolonged administration of BPs is a risk factor for AFFs [9]. Together with the multiple logistic regression analysis results showing an association between all BPs and drug-induced fractures, fractures associated with the use of osteoporosis BPs are more likely to be osteoclast-related fractures such as AFFs. Caution may also be necessary for BPs used in malignancy. Denosumab, an anti-RANKL antibody, was plotted near the osteoporosis BPs. AFF [30] and MRONJ [31] also occur with denosumab use. Our findings suggest that denosumab increases the risk of AFFs and jaw fractures via effects on osteoclasts and that the risk is comparable to that of BPs.

We observed that the first and second principal components of prednisolone both correlated negatively, suggesting that its use may contribute to drug-induced fractures through an osteoclast-mediated mechanism, although weaker than that of BPs and denosumab. In contrast, corticosteroids are reported to cause osteoporosis by affecting osteoblasts [58]; however, osteoblasts affect osteoclasts and vice versa [59], such that corticosteroid use may affect osteoclasts through osteoblasts.

Many drugs investigated in this study correlated positively with the second principal component, suggesting that factors other than osteoclasts may be involved in drug-induced fractures. Anastrozole correlated positively in both the first and second principal components; it is known to decrease bone mineral density and increase the risk of osteoporosis by suppressing estrogen production through aromatase inhibition [60]. Zolpidem correlated negatively in the first principal component and positively in the second principal component; zolpidem is a short-acting nonbenzodiazepine drug that can cause fractures due to falls [61]. Our results confirm that these drugs are associated with non-osteoclast-mediated fracture. Therefore, the principal component analysis results of the present study suggest that drug-induced fractures occur through a variety of mechanisms but that BPs and denosumab cause AFFs, jaw fractures, and ulna fractures via osteoclast-mediated mechanisms.

### 3.4. Limitations

This study has several limitations. First, because the database contains information on adverse drug reactions based on spontaneous reports, the cases are limited to those recognized as adverse drug reactions. The total number of patients using each drug is not known in this study, and therefore, a true assessment of adverse events cannot be made. Second, some of the JADER data is incomplete and has errors in letters and numbers. Thus, adverse events and drug names were corrected whenever possible, and patient background was evaluated using BMI. Third, when multiple drugs are administered, it is difficult to identify the specific cause of the adverse events. Furthermore, in the JADER data, fatal side effects are verified by the Pharmaceuticals and Medical Devices Agency, but other adverse events are based on the judgment of the reporter, meaning that the use of JADER data has several drawbacks. On the other hand, JADER is the largest database of spontaneously reported adverse drug reactions in Japan, and the information on adverse drug reactions obtained from JADER is expected to reflect not only unique pharmacological and pharmacokinetic characteristics but also prescription and usage conditions. Therefore, JADER is an excellent tool for inductively understanding adverse drug reactions and is used in many research fields.

## 4. Materials and Methods

### 4.1. JADER Database and the Selection of Data for Analysis

The JADER is the largest Japanese database of information that can be used to identify trends in the occurrence of adverse drug reactions. The JADER is a voluntary reporting database made publicly available by the Pharmaceuticals and Medical Devices Agency [62]. This study analyzes JADER data from 662,885 adverse drug reaction reports filed from 1 April 2004 to 31 December 2020 (Figure 1). The JADER case reports are categorized into four tables: DRUG (e.g., drug name, causal relationship), REAC (e.g., adverse events, outcomes), DEMO (patient demographic information such as sex, age, weight), and HIST (e.g., medical history, primary disease). In this study, data in the DRUG, REAC, and DEMO tables were used. Based on their level of involvement in the adverse events, drugs in the DRUG table were assigned to one of three categories: suspected drugs, concomitant drugs, and interactions; this study used only the data for “suspected drugs”. The world health organization drug classification ATC code was added to each drug for the purpose of tabulating drug effects [63]. The adverse events in the REAC table are recorded by preferred terms from the International Council for Harmonisation of Technical Requirements for Pharmaceuticals for Human Use of Pharmaceutical Terms (Medical Dictionary for Regulatory Activities Japanese version 23.1 (MedDRA/J v23.1)) [64]. Duplicate cases in the DRUG and REAC tables were eliminated as reported by Hirooka et al. [7,65]. The DRUG, REAC, and DEMO tables were combined using identification numbers. The patient BMI for each case was calculated using data in the tables; to eliminate incompatible reports, cases involving patients with a BMI <10 (*n* = 3215) or >100 (*n* = 1292) were not included in the analysis (Figure 1). Drug-induced fractures were defined as those that included “fracture” in the listed adverse drug reaction.

### 4.2. Association between Patient Characteristics and Drug-Induced Fracture by Single Regression Analysis

Fracture cases were divided into “drug-induced” and “non-drug-induced” for comparison. Patient characteristics, including age, height, and weight, were treated as absolute numbers, and *p*-values were calculated using Wilcoxon’s rank sum test. Weight in the 60 kg range was converted to 65 kg, and age under 10 years was converted to 5 years. For comparing data according to sex, *p*-values were calculated using Fisher’s direct exact test. Each patient factor was analyzed only with data that did not contain missing values.

### 4.3. Association between Drug Use and Drug-Induced Fractures by Single Regression Analysis

**Figure 4:**
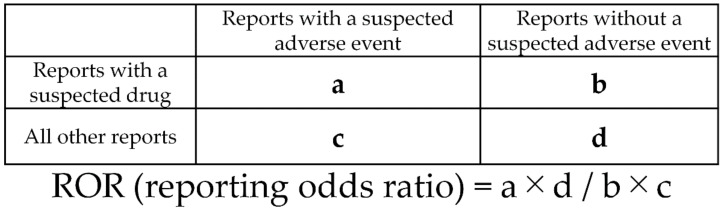
Cross-tabulation and formula used to calculate the ROR for an adverse event. The cross-tabulation is structured with reports for the suspected drug, all other reports, reports with an adverse event, and reports without an adverse event (a–d indicate the number of reports). This study shows the reporting odds ratio (ROR) as the odds ratio (OR).

Fracture cases were divided into “drug-induced” and “non-drug-induced” for comparison. The analysis included 4892 drugs reported as “suspected”. The relationship between each drug and the occurrence of drug-induced fractures was evaluated using the reported odds ratio (ROR) and Fisher’s direct exact test. First, a 2 × 2 contingency table for drugs and adverse events was constructed for all drugs (Figure 4). The 2 × 2 contingency tables were corrected by adding 0.5 to all cells (Haldane Anscombe 1/2 correction) [66,67]. Drugs with a ROR ≥1 and Fisher’s direct exact test *p* ≤ 0.05 were considered to be associated with drug-induced fractures. Next, a volcano plot consisting of the RORs and *p*-values of all drugs was constructed. A scatter plot was created using the natural logarithm of the ROR (lnOR) and the normal logarithm of the *p*-value (−log [P]). The scatter plot corresponds to the volcano plot, which is frequently used in the bioinformatics field to determine gene expression trends [68,69].

### 4.4. Associated between Patient Characteristics and Drug-Induced Fracture by Multiple Logistic Regression Analysis

Multiple logistic regression analysis was performed using the presence or absence of drug-induced fracture as the objective variable and patient background and drug factors, which showed significant differences in the previous section, as explanatory variables [70]. Multiple logistic regression analysis was performed using 675,785 reports, excluding missing reports with missing data.

### 4.5. Association between Drug Use and Drug-Induced Fracture Site by Principal Component Analysis

Principal component analysis was conducted to identify relationships between the site of drug-induced fracture and specific drugs. Principal component analysis is a method for determining the principal component by collapsing information from multiple dimensions, each of which has its own information [71,72]. Of the 90 drugs that were significantly associated with drug-induced fractures in multiple logistic regression analysis, the 25 drugs with more than 50 reports were analyzed. For adverse events, 22 adverse drug reactions with more than 70 reports in the drug-induced fracture group were analyzed. The RORs were transformed to the natural logarithm and subjected to principal component analysis by correlation matrix. Of the newly generated principal components, the first and second were used to interpret the characteristics of drugs and side effects.

### 4.6. Statistical Analysis

All analyses were performed using JMP Pro13.2.0 (SAS Institute Inc., NC, U.S.A.), and *p* < 0.05 was considered significant.

## 5. Conclusions

Analysis of drug-induced fractures in the JADER database revealed three new findings. First, fractures occurred with the use of many types of drugs associated with osteoporosis and falls, especially fractures of the femur and spine. Second, drug-induced fractures were associated with the use of 90 drugs and several patient characteristics. Drug groups, including bisphosphonates, denosumab, anti-HIV and other antiviral drugs, corticosteroids and sex hormone-related drugs, as well as sleep sedatives and dopaminergic drugs, were shown to have an effect on drug-induced fractures. Third, AFF, MRONJ, and ulnar fractures were associated with the use of bisphosphonates, denosumab, and corticosteroids via effects on osteoclasts. Other drugs were found to increase fracture risk without affecting osteoclasts. We hope that the findings of this study will contribute to the appropriate management of side effects by healthcare professionals.

## Figures and Tables

**Table 1:** Frequency of the 58 adverse events defined as drug-induced fractures.

Adverse Event	Reporting Times	Reporting Ratio (%)
Atypical femur fracture	1653	17.3%
Fracture	1550	16.3%
Femur fracture	1366	14.3%
Spinal compression fracture	1059	11.1%
Femoral neck fracture	619	6.5%
Compression fracture	355	3.7%
Rib fracture	261	2.7%
Lumbar vertebral fracture	217	2.3%
Humerus fracture	199	2.1%
Pelvic fracture	197	2.1%
Spinal fracture	142	1.5%
Pathological fracture	129	1.4%
Radius fracture	128	1.3%
Foot fracture	113	1.2%
Atypical fracture	111	1.2%
Upper limb fracture	106	1.1%
Jaw fracture	92	1.0%
Osteoporotic fracture	79	0.8%
Lower limb fracture	76	0.8%
Tibia fracture	76	0.8%
Cervical vertebral fracture	74	0.8%
Ulna fracture	74	0.8%
Clavicle fracture	67	0.7%
Ankle fracture	67	0.7%
Thoracic vertebral fracture	66	0.7%
Hip fracture	64	0.7%
Facial bones fracture	60	0.6%
Multiple fractures	55	0.6%
Hand fracture	50	0.5%
Wrist fracture	37	0.4%
Skull fracture	37	0.4%
Stress fracture	36	0.4%
Patella fracture	36	0.4%
Scapula fracture	28	0.3%
Fibula fracture	28	0.3%
Traumatic fracture	27	0.3%
Ilium fracture	26	0.3%
Sternal fracture	22	0.2%
Fractured sacrum	18	0.2%
Avulsion fracture	16	0.2%
Skull fractured base	14	0.1%
Comminuted fracture	13	0.1%
Fracture delayed union	12	0.1%
Forearm fracture	11	0.1%
Fracture nonunion	9	0.1%
Fracture pain	8	0.1%
Limb fracture	8	0.1%
Complicated fracture	8	0.1%
Open reduction of fracture	6	0.1%
Periprosthetic fracture	6	0.1%
Lisfranc fracture	5	0.1%
Internal fixation of fracture	4	<0.1%
Open fracture	3	<0.1%
Fractured coccyx	3	<0.1%
Acetabulum fracture	2	<0.1%
Epiphyseal fracture	1	<0.1%
Sacroiliac fracture	1	<0.1%
Fractured skull depressed	1	<0.1%

**Table 2:** Comparison of patient characteristics between drug-induced and non–drug-induced fractures.

Patient Characteristics	Drug-Induced Fracture (9531)	Non–Drug-Induced Fracture (1,675,323)	*p*-Value
Sex ^#^ (male; female)	2530; 6533 (9063)	837,777; 785,685 (1,623,462)	<0.001 ^###^
Age ^†^	69.1 ± 17.4 (8239)	59.5 ± 21.5 (1,565,208)	<0.001 ***
Height (cm) ^†^	154.0 ± 12.6 (2369)	157.2 ± 18.3 (703,033)	<0.001 ***
Weight (kg) ^†^	52.1 ± 13.7 (2721)	54.5 ± 16.3 (821,264)	<0.001 ***
BMI ^†^	22.2 ± 4.5 (2286)	21.9 ± 4.5 (680,578)	0.010 **

BMI, body mass index. Some values were missing for each variable; analyses were performed using data after eliminating these records. The numbers in parentheses are the numbers of cases used in the analyses. ^#^ Fisher’s exact test; ^†^ Wilcoxon signed-rank test; ** *p* < 0.05; ^###^, *** *p* < 0.001.

**Table 3:** Multiple logistic regression analysis of fracture risk according to drug use and patient characteristics (*n* = 675,785).

Risk Factor	Drug Class	Odds Ratio	95% Confidence Interval	*p*-Value
etidronic acid	drugs affecting bone structure and mineralization	189.01	73.21–487.95	<0.001 **
trientine	other alimentary tract and metabolism products	164.48	19.57–1382.36	<0.001 **
dolutegravir	direct acting antivirals	151.14	63.90–357.50	<0.001 **
abacavir	direct acting antivirals	123.71	37.41–409.03	<0.001 **
prednisolone sodium phosphate	corticosteroids	106.92	12.67–902.28	<0.001 **
alendronic acid	drugs affecting bone structure and mineralization	78.13	66.18–92.25	<0.001 **
adefovir dipivoxil	direct acting antivirals	71.83	51.25–100.68	<0.001 **
laninamivir	direct acting antivirals	63.91	33.37–122.41	<0.001 **
abacavir sulfate and lamivudine	direct acting antivirals	60.8	18.93–195.25	<0.001 **
minodronic acid hydrate	drugs affecting bone structure and mineralization	59.15	44.62–78.41	<0.001 **
risedronic acid	drugs affecting bone structure and mineralization	59.14	46.46–75.29	<0.001 **
radium (223Ra) dichloride	other therapeutic radiopharmaceuticals	53.77	31.62–91.44	<0.001 **
cepharanthine	isoquinoline alkaloids	51.53	12.01–221.06	<0.001 **
tetrabenazine	other nervous system drugs	45.02	21.90–92.55	<0.001 **
denosumab	drugs affecting bone structure and mineralization	44.12	35.96–54.14	<0.001 **
L-aspartate potassium	potassium	40.9	17.75–94.25	<0.001 **
nafarelin	hypothalamic hormones	36.81	4.90–276.58	0.001 *
ropinirole	dopaminergic agents	33.28	14.49–76.42	<0.001 **
lamivudine	direct acting antivirals	33.03	18.86–57.86	<0.001 **
ibandronic acid	drugs affecting bone structure and mineralization	32.76	20.59–52.12	<0.001 **
clostridium butyricum	antidiarrheal microorganisms	30.21	14.05–64.95	<0.001 **
interferon beta-1a	immunostimulants	30.19	4.07–224.02	0.001 *
anastrozole	hormone antagonists and related agents	29.22	17.50–48.77	<0.001 **
trelagliptin succinate	blood glucose lowering drugs, excl. insulins	28.21	12.31–64.64	<0.001 **
incadronate disodium hydrate	drugs affecting bone structure and mineralization	27.8	6.63–116.61	<0.001 **
emtricitabine and tenofovir disoproxil	direct acting antivirals	26.95	3.68–197.42	0.001 *
teneligliptin and canagliflozin	blood glucose lowering drugs, excl. insulins	26.36	8.25–84.31	<0.001 **
tafamidis	other nervous system drugs	24.85	7.84–78.76	<0.001 **
methylphenidate	psychostimulants, agents used for adhd and nootropics	23.69	7.48–74.98	<0.001 **
zoledronic acid	drugs affecting bone structure and mineralization	23.67	18.27–30.67	<0.001 **
romosozumab	drugs affecting bone structure and mineralization	23.64	12.80–43.66	<0.001 **
ritonavir	direct acting antivirals	22.23	3.05–161.95	0.002 *
calcium L-aspartate hydrate	calcium	20.87	7.60–57.35	<0.001 **
somatropin	anterior pituitary lobe hormones and analogues	19.05	8.40–43.20	<0.001 **
pamidronic acid	drugs affecting bone structure and mineralization	18.98	10.05–35.85	<0.001 **
etelcalcetide	anti-parathyroid agents	18.95	6.98–51.44	<0.001 **
exemestane	hormone antagonists and related agents	16.66	7.80–35.57	<0.001 **
memantine	anti-dementia drugs	16.11	9.55–27.17	<0.001 **
rasagiline	dopaminergic agents	15.96	5.86–43.44	<0.001 **
leuprorelin	hormones and related agents	14.94	9.43–23.67	<0.001 **
certolizumab pegol	immunosuppressants	14.42	7.12–29.23	<0.001 **
tofacitinib	immunosuppressants	14.09	10.43–19.04	<0.001 **
abiraterone	hormone antagonists and related agents	14.05	7.68–25.71	<0.001 **
tenofovir alafenamide	direct acting antivirals	13.95	3.43–56.67	<0.001 **
perampanel	antiepileptics	13.87	3.41–56.45	<0.001 **
methylprednisolone	corticosteroids	13.55	8.83–20.80	<0.001 **
teriparatide	parathyroid hormones and analogues	13.36	5.90–30.25	<0.001 **
goserelin	hormones and related agents	13.22	5.88–29.75	<0.001 **
paliperidone	antipsychotics	12.61	4.67–34.01	<0.001 **
entacapone	dopaminergic agents	12.6	4.00–39.70	<0.001 **
letrozole	hormone antagonists and related agents	12.43	6.38–24.23	<0.001 **
tramadol	opioids	12.29	6.32–23.91	<0.001 **
raloxifene	other sex hormones and modulators of the genital system	11.99	6.71–21.41	<0.001 **
eszopiclone	hypnotics and sedatives	11.07	2.72–45.00	0.001 *
istradefylline	other antiparkinson drugs	11	2.70–44.75	0.001 *
fluticasone furoate and vilanterol trifenatate	adrenergics, inhalants	10.56	1.46–76.28	0.019 *
zopiclone	hypnotics and sedatives	10.17	4.52–22.87	<0.001 **
aripiprazole hydrate	antipsychotics	9.89	2.44–40.08	0.001 *
suvorexant	hypnotics and sedatives	9.75	4.01–23.69	<0.001 **
hydrocortisone	corticosteroids	8.79	2.17–35.60	0.002 *
levodopa and benserazide hydrochloride	dopaminergic agents	8.51	2.71–26.72	<0.001 **
pioglitazone	blood glucose lowering drugs, excl. insulins	8.43	5.19–13.70	<0.001 **
rotigotine	dopaminergic agents	8.34	1.15–60.30	<0.001 **
alogliptin	blood glucose lowering drugs, excl. insulins	8.13	3.84–17.22	<0.001 **
golimumab	immunosuppressants	8.09	4.75–13.78	<0.001 **
prednisolone	corticosteroids	8.08	6.73–9.70	<0.001 **
pramipexole	dopaminergic agents	7.93	3.27–19.23	<0.001 **
enzalutamide	hormone antagonists and related agents	7.64	3.78–15.42	<0.001 **
teriparatide acetate	parathyroid hormones and analogues	7.64	4.18–13.96	<0.001 **
cinacalcet	anti-parathyroid agents	6.9	2.20–21.63	0.001 *
buprenorphine	opioids	6.83	1.69–27.64	0.007 *
salmeterol xinafoate and fluticasone propionate	corticosteroids	6.64	1.65–26.78	0.008 *
bazedoxifene	other sex hormones and modulators of the genital system	6.57	2.09–20.62	0.001 *
betamethasone	corticosteroids	6.46	2.67–15.64	<0.001 **
ledipasvir acetonate and sofosbuvir	direct acting antivirals	6.41	2.86–14.40	<0.001 **
eldecalcitol	vitamin a and d, incl. combinations of the two	6.39	3.67–11.12	<0.001 **
galantamine	anti-dementia drugs	6.36	2.83–14.31	<0.001 **
alprazolam	anxiolytics	6.24	2.32–16.76	<0.001 **
rivastigmine	anti-dementia drugs	5.86	2.17–15.77	0.001 *
zolpidem	hypnotics and sedatives	5.85	3.02–11.31	<0.001 **
urapidil	antiadrenergic agents, peripherally acting	5.74	0.80–41.38	0.083
brotizolam	hypnotics and sedatives	5.67	2.68–11.97	<0.001 **
pregabalin	antiepileptics	5.55	3.85–8.00	<0.001 **
sofosbuvir	direct acting antivirals	5	2.06–12.09	<0.001 **
carbidopa hydrate and levodopa	dopaminergic agents	4.29	1.38–13.41	0.012 *
donepezil	anti-dementia drugs	4.15	1.96–8.77	<0.001 **
tramadol hydrochloride and acetaminophen	opioids	3.85	1.59–9.31	0.003 *
mirogabalin besylate	other analgesics and antipyretics	3.77	0.53–27.07	0.187
darbepoetin alfa	other antianemic preparations	3.75	1.40–10.04	0.009 *
alfacalcidol	vitamin a and d, incl. combinations of the two	3.48	1.30–9.34	0.013 *
duloxetine	antidepressants	3.09	1.28–7.47	0.012 *
tocilizumab	immunosuppressants	3.06	1.77–5.30	<0.001 **
methoxy polyethylene glycol-epoetin beta	other antianemic preparations	2.73	0.68–10.98	0.157
clozapine	antipsychotics	2.34	0.58–9.41	0.23
ramelteon	hypnotics and sedatives	2.32	0.32–16.57	0.402
aliskiren	other agents acting on the renin-angiotensin system	2.28	0.32–16.31	0.41
paliperidone palmitate	antipsychotics	2.27	0.32–16.17	0.414
abatacept	immunosuppressants	2.25	0.72–7.02	0.161
quetiapine	antipsychotics	1.89	0.61–5.87	0.274
mirabegron	urologicals	1.64	0.23–11.72	0.62
etanercept	immunosuppressants	1.56	0.74–3.29	0.24
ixazomib	other antineoplastic agents	1.18	0.29–4.72	0.818
female	―	2.05	1.86–2.26	<0.001 **
Unit Odds Ratio				
Risk Factor		Odds Ratio	95% Confidence Interval	*p*-Value
age	―	1.02	1.01–1.02	<0.001 **
BMI	―	1.02	1.01–1.03	<0.001 **
Range Odds Ratio				
Risk Factor		Odds Ratio	95% Confidence Interval	*p*-Value
age	―	5.59	4.21–7.43	<0.001 **
BMI	―	5.49	2.64–11.45	<0.001 **

BMI, body mass index. Analyses were performed after eliminating records with missing data. * *p* < 0.05; ** *p* < 0.001.

## Data Availability

Data is contained within article and Supplementary Materials.

## Supplementary Materials

The following are available online at https://www.mdpi.com/article/10.3390/ph14121299/s1, Table S1: Fisher’s exact test results according to the presence or absence of drug-induced fracture.
